# Pulmonary neuroendocrine cells: physiology, tissue homeostasis and disease

**DOI:** 10.1242/dmm.046920

**Published:** 2020-12-21

**Authors:** Masafumi Noguchi, Kana T. Furukawa, Mitsuru Morimoto

**Affiliations:** 1 Laboratory for Lung Development and Regeneration, RIKEN Centre for Biosystems Dynamics Research, Kobe 650-0047, Japan; 2 Department of Biology, University of Padova, Via U. Bassi 58B, 35121 Padova, Italy; Veneto Institute of Molecular Medicine, Via Orus 2, 35129 Padova, Italy

**Keywords:** Development, Lung, Neuroendocrine, Regeneration, Respiratory diseases, Vagal nerves

## Abstract

Mammalian lungs have the ability to recognize external environments by sensing different compounds in inhaled air. Pulmonary neuroendocrine cells (PNECs) are rare, multi-functional epithelial cells currently garnering attention as intrapulmonary sensors; PNECs can detect hypoxic conditions through chemoreception. Because PNEC overactivation has been reported in patients suffering from respiratory diseases – such as asthma, chronic obstructive pulmonary disease, bronchopulmonary dysplasia and other congenital diseases – an improved understanding of the fundamental characteristics of PNECs is becoming crucial in pulmonary biology and pathology. During the past decade, murine genetics and disease models revealed the involvement of PNECs in lung ventilation dynamics, mechanosensing and the type 2 immune responses. Single-cell RNA sequencing further unveiled heterogeneous gene expression profiles in the PNEC population and revealed that a small number of PNECs undergo reprogramming during regeneration. Aberrant large clusters of PNECs have been observed in neuroendocrine tumors, including small-cell lung cancer (SCLC). Modern innovation of imaging analyses has enabled the discovery of dynamic migratory behaviors of PNECs during airway development, perhaps relating to SCLC malignancy. This Review summarizes the findings from research on PNECs, along with novel knowledge about their function. In addition, it thoroughly addresses the relevant questions concerning the molecular pathology of pulmonary diseases and related therapeutic approaches.

## Abstract (precis)

**Summary:** This Review highlights the physiological relevance of pulmonary neuroendocrine cells, rare airway epithelial cells that form intrapulmonary sensory organs, abnormalities of which are associated with several pulmonary disorders, such as asthma and lung cancer.

## Introduction

Every time we take a breath to convey oxygen (O_2_) into our body, our respiratory tissues are exposed to external air, which contains ambient aerosols along with a multitude of pathogens, allergens and pollutants. The lung senses and recognizes these risk factors and activates defense reactions via immune signaling and neuronal circuitry. However, the cell type capable of feeling such chemical and mechanical stresses has remained unknown for a long time. Pulmonary neuroendocrine cells (PNECs) are one of the epithelial cell types lining large and small airways that form a tiny cellular population (0.4% of total airway epithelial cells) and are evolutionarily preserved among air-breathing vertebrates. Their chemosensitivity features have previously been demonstrated. Furthermore, PNECs are starting to be considered as the most important O_2_- and chemical/physical stimuli-sensing epithelial cells in the airways (Garg et al., 2019).

**Box 1. Glossary**

***Ascl1*:** a gene encoding achaete-scute homolog 1, a neurogenic transcription factor of the basic helix-loop-helix (bHLH) family that plays key roles in neuronal commitment.

**Calbindin-D (28k):** a cytosolic Ca^2+^-binding protein that controls synaptic Ca^2+^ dynamics in the neuron.

**Calcitonin gene-related peptide (CGRP):** a 37-amino acid neuropeptide that modulates metabolism, inflammatory responses and blood pressure.

**Chromaffin cells:** neuroendocrine cells in the adrenal medulla, releasing neurohormones into the blood. The word ‘chromaffin’ is a portmanteau of ‘chromium affinity’, as these cells can be stained with chromium salts for histological analysis.

**Chromogranin-A** **(CgA):** a prohormone playing a role in the biogenesis of secretory dense-core vesicles (see below) and hormone sequestration in endocrine, neuroendocrine and neuronal cells.

**Club cells:** dome-shaped secretory cells synthesizing surfactant and mucin found throughout the tracheobronchial airway epithelium.

**Dense-core vesicles (DCVs):** membrane-bound organelles in which endocrine cells package diverse cargo, such as neurotransmitters and other molecules.

**Emphysema:** a pulmonary disease that causes shortness of breath in which alveolar-capillary units are damaged and the surrounding supporting tissue is lost.

**Gastrin-releasing peptides (GRPs)/bombesin-like peptides (BLPs):** GRP is the mammalian homolog of bombesin, a tetradecapeptide initially identified in the skin of European amphibians, which includes the C-terminal ten-amino acid sequences similar to bombesin. It, like other bombesin-like peptides (BLPs), affects the smooth muscle cells’ contractile function and potently induces bronchoconstriction via the GRP receptor.

**Goblet cells:** goblet-shaped cells producing mucin MUC5AC in the respiratory epithelium.

**Hering–Breuer mechanoreflex:** a reflex of apnea preventing lung over-inflation that is triggered when pulmonary mechanoreceptors respond to excessive stretching.

**Hes1:** a basic helix-loop-helix family transcription factor that is the key downstream effector for Notch signaling pathway.

**Hypoxia-inducible factor 1** **(HIF-1):** a transcription factor playing an integral role in the response to low oxygen concentrations.

**Lipopolysaccharide (LPS):** a glycolipid of the outer membrane of Gram^–^ bacteria. Intratracheal LPS instillation induces intrapulmonary inflammation in mice.

**Lysine-specific histone demethylase 1 (LSD1):** a component of the nucleosome remodeling and deacetylating (NuRD) complex, although it silences genes by functioning as a histone demethylase. Its binding to a region within the *NOTCH1* gene is associated with increased histone-3 lysine-27 acetylation and results in NOTCH1 silencing.

**NKX2.1:** a homeodomain transcriptional factor expressed in thyroid, lung and brain, which regulates the gene expression involved in initial specification of the organs.

**P2X_2/3_:** purinergic receptor channels mediating nociception in primary sensory neurons.

**P2Y purinoceptor 1** **(P2RY1):** a G-coupled protein receptor responding to purine and pyrimidine nucleotides.

**Piezo2:** a mechanosensitive cation channel playing a role in transmitting tactile stimuli.

**Polycomb repressive complex 2 (PRC2):** an enzyme that catalyzes histone H3K27 trimethylation for the epigenetic silencing of genes in development and cancer.

**Slit–Roundabout (ROBO) signaling:** the secreted ligand Slit binds to the cell surface receptor Roundabout (ROBO) to transmit migratory cues by regulating cell adhesion and cytoskeletal organization.

**Transforming growth factor β1** **(TGFβ1)–Alk5 signaling:** TGFβ1 is a pleiotropic growth factor, controlling cell proliferation, differentiation and apoptosis. Alk5 is the major type 1 receptor for TGFβ ligands.

**Transient receptor potential cation channel subfamily C member 5 (Trpc5):** a nonselective cation channel belonging to the transient receptor potential channels, activated by G-protein-coupled receptors.

**Type II alveolar cells:** an epithelial cell type in alveoli, which expresses proteins associated with surfactant production and its secretion.

**Vesicular glutamate transporters (VGLUTs):** transporters driven by a proton gradient to translocate the neurotransmitter glutamate from the cytosol into synaptic vesicles.

PNECs were first described as ‘helle zellen’ (‘bright cells’ in German) by Dr Fröhlich in 1949 (Fröhlich, 1949). Feyrter (1954) further confirmed the presence of secretory dense-core vesicles (DCVs; see Glossary, Box 1) and established the concept of the diffuse neuroendocrine system, suggesting that the human endocrine system might include scattered cells presenting as either isolated cells or aggregates. The endocrine system might present not only in ductal tissues (e.g. pancreas) but also diffused in the mucous columnar epithelia of the inner and outer surfaces of the human body (e.g. gastrointestinal tract and skin), and PNECs have been classified as part of the diffuse neuroendocrine system (Modlin et al., 2006; Feyrter, 1938).

In fact, PNECs appear either as solitary cells or clustered masses in the airway epithelium. Clustered PNECs – known as neuroepithelial bodies (NEBs) – are typically located next to airway bifurcations in a stereotypic fashion (Kuo and Krasnow, 2015; Noguchi et al., 2015) and frequently associate with intraepithelial nerve fibers. The anatomical features of NEBs reflect their unique role as the sensory component of the lung. Moreover, alterations in their histological structure and physiological functions occur in human pathology, particularly during lung cancer and asthma. NEBs sense such environments and communicate with the immune and nervous systems. If the lung is injured by chemical insults or viral infection, the airway epithelium boots this backup system to deal with the injuries. Further, PNECs also have stem cell properties, as they seem to play an important role in the regenerative response to severe airway damage (Hogan et al., 2014). Therefore, PNECs and NEBs are key players at the interface between the respiratory system and the external environment.

The pathophysiological significance of PNECs has been questioned since their discovery five decades ago. For the past two decades, however, cutting-edge technologies have shed new light on their roles, such as asthmatic-response amplifiers, tissue regeneration contributors and cells of origin of lung cancer. Although the PNEC population in the lung is small, PNEC activity affects several important processes in the respiratory system. In this Review, we summarize the key knowledge from the existing literature and delineate new paradigms in the biology and pathology of PNECs.

## Anatomy of PNECs

### Distribution and population in the airways

Boers et al. (1996) described the distribution and populations of PNECs in adult humans: chromogranin-A (CgA)^+^ (Box 1) PNECs account for 0.41% of all epithelial cells in the conducting airway, but are absent from the alveoli. Recent single-cell RNA sequencing (scRNA-seq) analyses found that PNECs account for 0.01% of all lung cells (Travaglini et al., 2020). Owing to the extensive size of the human lung relative to the small amount of PNECs, determining their lung-wide distribution is rather difficult. Therefore, small mammals – including mice – represent ideal models for obtaining the entire picture of PNEC distribution throughout the tracheobronchial tree, mostly owing to the size advantage for imaging. As revealed by scanning electron microscopy, NEBs form crater-like pits, which are aligned with microvilli and exposed to the airway (Cutz et al., 1978).

**Fig. 1.**
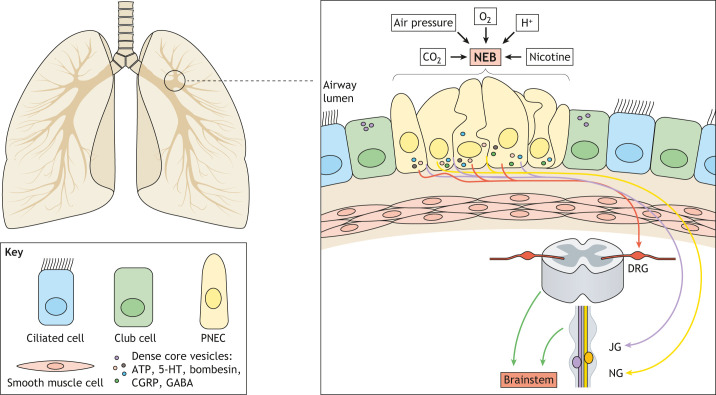
**Schematic** **representation of pulmonary neuroendocrine cells (PNECs), neuroepithelial bodies (NEBs) and their innervation in the airway.** In the mammalian lung, PNECs (yellow) localize at airway bifurcation sites (in the circled area and illustrated on the right), forming small clusters called NEBs. The NEB interacts with sensory nerve terminals, with myelinated afferent nerves (yellow and purple) branching and protruding into the NEB. The other sensory nerve (orange) comprises unmyelinated non-vagal immunoreactive nerve fibers originating from the dorsal root ganglia (DRG). Their axons enter the brain and transmit sensory information to the brainstem (green arrows). NEBs can sense CO_2_, air pressure, O_2_, H^+^ ions and nicotine, and activate reactions. ATP, adenosine triphosphate; CGRP, calcitonin gene-related peptide; GABA, gamma-aminobutyric acid; JG, jugular ganglion; NG, nodose ganglion; 5-HT, serotonin.

NEBs frequently populate diametrically opposed positions to the bifurcation points of branching airways (Avadhanam et al., 1997; Kuo and Krasnow, 2015; Noguchi et al., 2015) (Fig. 1). NEBs at branching points are referred to as ‘nodal’ NEBs, whereas NEBs in inter-bifurcation regions are referred to as ‘internodal’. During development, nodal NEBs overcome internodal NEBs; moreover, NEBs grow centrifugally, from proximal to distal bronchi. Three-dimensional (3D) geometric analyses showed nodal NEBs at stereotypic positions in the airway branching structures (Noguchi et al., 2015). In rats, the distribution pattern of NEBs, as well as their absolute number, remains unchanged after birth (Avadhanam et al., 1997). This peculiar distribution of NEBs may be optimal for sensing hypoxic conditions and allergens in the airways; nonetheless, further studies are still required to understand its significance, as well as the functional differences between nodal and internodal NEBs.

### PNEC innervation

In 1972, Lauweryns and Peuskens identified innervated PNECs within the intrapulmonary airway epithelium of human infants (Lauweryns and Peuskens, 1972). Further detailed imaging revealed that various types of sensory (afferent) and motor (efferent) nerve fibers connect to PNECs (Lauweryns et al., 1985) (Fig. 1). NEBs are predominantly innervated by vagal nerve fibers originating from cell bodies located in the nodose ganglion, mainly involved in visceral perception (Adriaensen et al., 1998). Several different types of vagal nerves interact with NEBs, including Na^+^/K^+^ ATPase^+^, VGLUT^+^, calbindin-D (28k)^+^ or P2X_2/3_^+^ (also known as P2RX2/3^+^) (Box 1) nerves (Adriaensen et al., 2006). These myelinated afferent nerves lose their sheaths right next to NEBs and then branch and protrude into the epithelium (Brouns et al., 2000, 2003). Conversely, unmyelinated non-vagal calcitonin gene-related peptide (CGRP)^+^ (also known as CALCA^+^) (Box 1) nerve fibers, which originate from dorsal root ganglia T1 to T6, make contact with the basal pole of pulmonary NEBs (Brouns et al., 2003; Haller, 1992). Calbindin-D (28k)^+^ and CGRP^+^ nerve fibers often make contact with the same NEBs. Unlike calbindin-D (28k)^+^ nerves, CGRP^+^ nerves express vanilloid receptor subtype 1 and respond to capsaicin, suggesting their C-nociceptive nature (Brouns et al., 2003; Baron, 2000). Chang et al. (2015) identified that P2Y purinoceptor 1 (P2RY1; Box 1) is also expressed in the vagal sensory neurons associated with PNECs. The cell bodies of P2RY1^+^ nerves reside in the nodose/jugular ganglia; their axons enter the brain and target the lateral solitary tract to transmit sensory information to the brainstem nucleus of the dorsal respiratory group, which regulates breathing (Speck and Feldman, 1982). Activation of P2RY1^+^ neurons activates reflective airway defense mechanisms, such as apnea, vocal fold adduction, swallowing and expiratory reflexes (Prescott et al., 2020). The functional relevance of NEBs and P2RY1^+^ neurons is an intriguing topic for future research. Defining the role of NEB–P2RY1^+^ communication could lead to a more complete understanding of the link between airway status and physiological reflexes.

NEB innervation increases with advancing gestation, reaching a plateau after birth (Pan et al., 2004). How do NEBs guide the afferent nerve fibers during development? Both solitary and clustered PNECs are innervated in the human lung (Brouns et al., 2003). Because solitary PNECs are still innervated in mutant mice that fail to form NEBs, clustering seems dispensable for innervation (Branchfield et al., 2016). Subsequent research showed that nerve tracks remain close to the epithelium in PNEC-depleted lungs (Sui et al., 2018). PNECs attract nerve terminals and induce their intraepithelial protrusion. Barrios et al. (2017) reported that PNECs express neurotrophin 4 (NT4; also known as NTF4), while innervating nerves express its cognate receptor TrkB (also known as NTRK2). NT4 plays a role in the formation of nerve contacts to the basal side of NEBs and their penetration therein during development; however, NT4 ablation does not entirely abolish innervation, suggesting that additional unknown factors contribute to this synapse formation. Aside from identifying these factors, genetic modulation of neuro-PNEC junctions would help to characterize the physiological functions of PNEC innervation.

## PNECs as sensory transducers

In the mid-20th century, the nature of intrapulmonary chemoreceptors was yet to be determined, even though physiological observation anticipated their existence (Dawes and Comroe, 1954) and indicated that hypoxia evokes pulmonary vasoconstriction, possibly via the monoamine neurotransmitter serotonin (5-HT) (Duke, 1951; Sjoerdsma, 1959). Moreover, a series of morphological observations by Lauweryns and Cokelaere (1973) identified structural similarities between NEBs and chemoreceptors in other tissues, such as taste buds and carotid bodies. Furthermore, PNECs express and secrete 5-HT in response to hypoxia (Lauweryns and Cokelaere, 1973; Lauweryns et al., 1977). Recent research further showed that PNECs can respond not only to hypoxia but also to several environmental stimuli and mechanical forces (Cutz et al., 2013). Here, we describe the physiological significance of PNECs as a sensory component of the lung.

### Oxygen sensing

Pulmonary tissue senses O_2_ in the inhaled air to control breathing rate via the central nervous system (Dawes and Comroe, 1954); this homeostatic response equilibrates O_2_ availability in different environments. For instance, when humans get a workout at high altitude, where air pressure is relatively low, their ventilation frequency increases to uptake more O_2_ into the lungs (West et al., 1983). In 1993, Youngson et al. reported that PNECs in NEBs express an O_2_-sensing complex consisting of an NADPH oxidase coupled to an O_2_-sensitive K^+^ channel in the plasma membrane (Youngson et al., 1993). They showed that the K^+^ channels on PNECs close down in hypoxic conditions, while voltage-sensitive Ca^2+^ channels open up to facilitate the influx of extracellular Ca^2+^, leading to Ca^2+^-dependent exocytosis of DCVs (Cutz et al., 2013). Release of DCV cargo affects the physiological functions of lung tissues through direct or indirect interaction via vagal afferent and central nerves (Youngson et al., 1993; Wang et al., 1996; Cutz and Jackson, 1985) (Fig. 1).

Hypoxia triggers 5-HT release from NEBs, which occurs within the physiological range expected in the airway [oxygen partial pressure (PO_2_), ∼95 mmHg] (Fu et al., 2002). 5-HT induces vasoconstriction of large and small muscular pulmonary arteries (MacLean et al., 1996; Morecroft et al., 1999). Thus, PNECs link hypoxia and the serotonergic system to modulate pulmonary homeostasis. By contrast, CGRP is a potent vasodilator (Brain et al., 1985). Although CGRP is persistently secreted during normoxia to maintain vascular smooth muscle contraction, hypoxia depletes CGRP from NEBs, eventually reducing the pressor response of pulmonary vasculature (Tjen et al., 1998). In summary, PNECs may coordinate blood flow in the lung by regulating the secretion of these reciprocally bioactive peptides.

The peculiar positioning of NEBs at airway bifurcation points seems to relate to the structural benefits of rapid sensing of O_2_-level changes. The larger NEBs located next to the proximal branching points of the proximal airway respond to hypoxia quicker than the carotid body, which senses alterations in O_2_ levels in the blood. In future studies, analyzing genetically modified mice with altered NEB distribution could unveil the physiological significance of NEBs for O_2_ sensing in detail.

### Nicotine sensing

PNEC hyperplasia has been reported in smoking-associated lung disorders, including chronic obstructive pulmonary disease (COPD) and asthma. Nicotine inhalation via cigarette and e-cigarette smoking promotes pulmonary edema and lung damage, along with abnormal leukocyte increases, leading to adverse effects in the lungs and the entire body (Ahmad et al., 2019). Nicotine is an agonist for nicotinic acetylcholine receptors (nAChRs), which physiologically respond to the neurotransmitter acetylcholine. Prenatal nicotine exposure increases NEB abundance in primate models (Fu and Spindel, 2009). How do PNECs sense nicotine exposure? Does nicotine-triggered functional alteration of PNECs link to pulmonary diseases?

PNECs express functional nAChRs; similarly to hypoxia, nicotine exposure suppresses the O_2_-sensitive A-type K^+^ channel, evoking an excitatory inward current (Sartelet et al., 2008; Fu et al., 2007). The excited NEBs secrete 5-HT through the a7-nAChR pathway (Schuller et al., 2003). These findings imply that nicotine induces pulmonary hypertension, potentially via enhanced 5-HT secretion from hyperplastic PNECs. Moreover, intravenous nicotine injections evoke reflex apnea in the expiratory position in cats and dogs, resembling the P2RY1 reflex (Domaye, 1955; Takasaki, 1956). The involvement of NEB–P2RY1^+^ neuron communication in nicotine-induced chemoreflexes is an attractive topic for future research. Nicotine-induced a7-nAChR signaling cascades regulate cancer-associated features, including cell proliferation (Hajiasgharzadeh et al., 2019). Thus, their activation could lead to the aberrant PNEC hyperplasia found in smoking-associated lung diseases. Because 97% of small-cell lung cancer (SCLC) patients have a history of smoking (Pesch et al., 2012), nAChR signaling in SCLC development could be another relevant research topic.

### Mechanosensing

Ventilation dynamics generate mechanical forces in the lung epithelium. Computational simulations have demonstrated that branching points – where nodal NEBs locate – are subjected to a higher air pressure than the surrounding epithelium (Sul et al., 2014). Fetal breathing – respiratory-like rhythmic activity – produces amniotic fluid flows into and out of the lung (Plosa and Guttentag, 2018). These amniotic fluid waves could also intermittently hit the branching points during development. Thus, fetal and post-natal breathing may mechanically stimulate nodal NEBs in every respiratory cycle.

Several studies have shown that mechanosensing is a PNEC function. Piezo2 and Trpc5 (Box 1) are expressed on PNECs and likely play key roles in mechanosensing. In addition, PNECs are innervated by Piezo2^+^ afferent fibers responsible for the Hering–Breuer mechanoreflex (Box 1) (Lembrechts et al., 2012; Nonomura et al., 2017). Cultured NEBs induce a selective, fast, reversible and reproducible Ca^2+^ rise in response to mechanical hypoosmotic stimuli (Lembrechts et al., 2012). Furthermore, mechanical stretch enhances 5-HT release from NEBs in rabbit models, further suggesting that PNECs might be mechanosensitive and are possibly capable of transducing mechanical information into neurotransmission (Pan et al., 2006). Another candidate mediator for PNEC mechanotransduction could be adenosine triphosphate (ATP), as known in various tissues (Kringelbach et al., 2015; Guan et al., 2018). In an *ex vivo* lung slice model, depolarization of PNECs with high K^+^ releases the ATP stored in DCVs (De Proost et al., 2009).

Interestingly, P2X_3_^+^ nerves are exclusively associated with ATP^+^ DCV-containing PNECs, which express the heteromeric purinergic P2X_2/3_ receptors (Brouns et al., 2000; Fu et al., 2004). PNEC-secreted ATP may bind to autoreceptors on PNECs, promoting its own secretion through an autocrine positive feedback loop.

Despite the above, the physiological significance of PNEC mechanosensing remains enigmatic. PNEC mechanotransduction might pace the diaphragmatic vertical movements through periodic neuronal activation to support smooth breathing. Moreover, the oversecretion of biological substances in response to artificial mechanical strain could contribute to ventilator-induced pediatric lung disease (see ‘PNECs in lung pathogenesis’ section).

### Signaling center in asthmatic response

Asthma is the most frequently diagnosed chronic disorder among children and adults, affecting 339 million people worldwide, and the number of patients is increasing every year (http://www.globalasthmareport.org/Global%20Asthma%20Report%202018.pdf). Asthma is a chronic disease of the innate and adaptive immune systems responding to allergens (Suarez et al., 2008; Pivniouk et al., 2020). Histologically, PNEC hyperplasia has been observed in asthmatic patients' lungs (Adriaensen and Timmermans, 2004; Sui et al., 2018). In addition, allergen challenges increase PNECs in animal models (Bousbaa and Fleury-Feith, 1991). Previous studies have implicated the immune-regulatory role of PNECs, and recent *in vivo* findings support this hypothesis.

Notably, Sui et al. (2018) demonstrated that endodermal *Ascl1* (Box 1)-knockout mice, which are PNEC deficient, lack the allergen-induced asthmatic response. Furthermore, intratracheal administration of CGRP and gamma aminobutyric acid (GABA) to these mutants recovers the immune response, including goblet cell (Box 1) hyperplasia. The CGRP produced by PNECs stimulates type 2 innate lymphoid cells, enriched at airway branching points, which triggers immune responses to allergens. PNECs secrete GABA, which promotes goblet cell differentiation (Sui et al., 2018). Furthermore, Branchfield et al. (2016) described an increase in neuropeptide secretion in *Robo1/2* mutant mice, in which PNECs fail to cluster, increasing immune responses and airway inflammation. Thus, PNEC clustering may act as a rheostat for the intrapulmonary immune system. These findings raise new questions concerning the detailed mechanisms by which allergens activate PNECs.

Inhaled glucocorticoids are widely used to suppress bronchial inflammation (Tripathi, 2016) and monoclonal antibodies to target type 2 asthma are currently emerging (Edris et al., 2019). Elucidating the links between PNECs and the type 2 immune responses could confirm that repurposing CGRP-targeted drugs is beneficial in inhibiting the asthmatic immune reaction. PNEC functions might be linked to Th-2 immune responses; therefore, CGRP-targeting drugs could be considered for inhibition of inflammation in asthma.

## Development of PNECs and NEBs

### Origin of PNECs

Like other neuroendocrine cells – such as chromaffin cells (Box 1) (Anderson and Axel, 1986) – PNECs are also believed to migrate from the neural crest and directly colonize organs. PNECs develop from embryonic lung epithelial progenitors. ^3^H-thymidine labeling during the late gestation of Syrian hamster provided experimental evidence supporting the endodermal origin of PNECs (Hoyt et al., 1990). Further lineage-tracing genetic studies using *Id2-CreERT2*, *Nkx2.1-Cre*, *Sox9-Cre*, *Sox17-CreERT2* and *Shh-Cre* murine strains demonstrated that PNECs arise from progenitors in the lung epithelium, especially progenitors located in lung buds (Rawlins et al., 2009; Song et al., 2012; Kuo and Krasnow, 2015; Hockman et al., 2017). Noteworthily, PNEC were not labeled in the lineage trace of the *Wnt1-Cre* mouse line, which was engineered to track neural crest development, excluding their origin from neural crest cells (Kuo and Krasnow, 2015).

Gill neuroendocrine cells (GNECs), found in fish, *Xenopus* and lampreys, are functionally and structurally similar to PNECs in ambient O_2_ sensing. PNECs and GNECs are of endodermal origin and not neural crest derived, supporting their shared evolutionary ancestry (Hockman et al., 2017).

### Differentiation from endoderm

PNEC differentiation from epithelial progenitors and NEB development co-occur during lung epithelial development (Fig. 1). In PNEC differentiation, Ascl1 and Hes1 (Box 1) play primary fate-selection roles. Ascl1 shows scattered solitary distribution in murine bronchi during early epithelial development from embryonic day (E)12.5, before other types of lung epithelial cells appear (Cutz et al., 1985; Kuo and Krasnow, 2015). Ascl1 also plays a crucial role in PNEC differentiation, because progenitor cells in *Ascl1*-null mice fail to differentiate into PNECs (Borges et al., 1997; Ito et al., 2000; Sui et al., 2018).

In the developing lung epithelium, unlike Ascl1-expressing PNECs, non-PNEC cells express the repressive Hes1. PNEC differentiation is enhanced in *Hes1*-deficient mice, suggesting that Hes1 limits the PNEC lineage (Ito et al., 2000; Noguchi et al., 2015). In the developing lung epithelium, PNECs express the Notch ligands Dll1, Dll4, Jag1 and Jag2 (Post et al., 2000; Tsao et al., 2009; Xu et al., 2010; Ouadah et al., 2019; Stupnikov et al., 2019), suggesting that they are Notch ligand-providing cells. In Notch1, Notch2 and Notch3 receptor triple-knockout mice, the number and size of NEBs are obviously increased, indicating that Notch receptors limit PNECs during lung development (Morimoto et al., 2012). By contrast, transgenic mice expressing the constitutively active Notch1 receptor have fewer PNECs (Shan et al., 2007; Morimoto et al., 2012). Moreover, Dll ligands – but not Jagged ones – are involved in the regulation of the size of NEBs (Stupnikov et al., 2019).

**Fig. 2.**
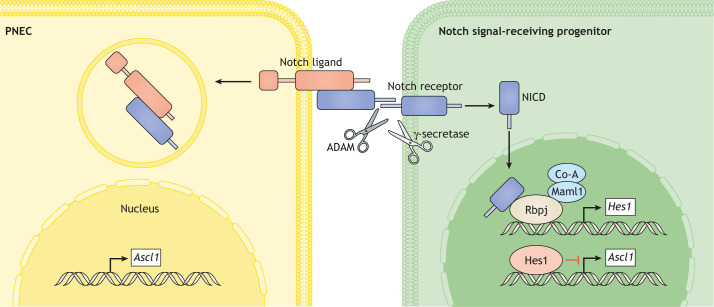
**Notch-mediated cell–cell interaction.** Notch is a type I transmembrane receptor that interacts with transmembrane ligands, such as Delta-like (Dll), on adjacent cells. Ligand binding leads to cleavage (by ADAM proteins and γ-secretase) and release of the Notch intracellular domain (NICD), which then moves to the nucleus to regulate transcriptional complexes containing the DNA-binding protein Rbpj. *Hes1* is an Rbpj-dependent Notch target gene that encodes a transcription factor that suppresses the expression of *Ascl1*, a key determinant of PNEC fate. PNECs are a Dll-expressing cell type and their neighboring cells are often Notch active.

These results indicate that PNEC fate selection from multipotent progenitor cells is governed by Notch-mediated lateral inhibition. In this model, naïve epithelial progenitors expressing both Notch receptor and ligand initially cover the entire epithelium of the primordial airway (Fig. 2). At the airway-branching stage, stochastic *Ascl1* upregulation in progenitor cells enhances Notch ligand expression and then activates Notch receptors on the neighboring progenitor cells. PNEC induction occurs proximo-distally as the distal-most lung buds elongate further. Notch signaling induces the expression of *Hes1*, which inhibits that of *Ascl1* in a cell-autonomous manner, resulting in a non-PNEC fate of the *Ascl1*^–^ cells. This Notch-mediated lateral inhibition results in solitary PNECs showing a salt-and-pepper pattern in the epithelium. *Hes1*-null developing lung epithelium shows multiple – but not solitary – PNECs, along with a disrupted salt-and-pepper pattern, supporting this model (Noguchi et al., 2015).

Although Notch signaling plays such an important role, loss of pan-Notch signaling retains *Hes1* expression and limits the reduction of PNECs, suggesting *Hes1* regulation by additional pathways aside from canonical Notch (Morimoto et al., 2010). Specifically, TGFβ1–Alk5 signaling (Box 1) is the most probable alternative (Xing et al., 2010). Although conditional Alk5 (also known as TGFBR1) knockout in the endoderm does not show any PNEC-related phenotype, *Hes1* expression becomes moderate in developing epithelial cells. TGFβ1–Alk5 signaling may ensure moderate expression of *Hes1* in lung epithelial cells, while Notch signaling may activate strong *Hes1* expression exclusively in the PNEC-surrounding cells. After PNEC specification via Ascl1, the zinc-finger protein Insm1 maintains *Ascl1* expression and thus progression towards differentiation to PNECs. Insm1 regulates the terminal differentiation of endocrine cells in several tissues. In *Insm1* mutant mice, PNECs fail to express the mature PNEC markers PGP9.5 (also known as UCHL1) and CGRP while retaining Ascl1 during their early development, suggesting that PNECs depend on Insm1 during their maturation phase following cell fate selection by Ascl1. Indeed, Ascl1 activates the expression of *Insm1*, and, subsequently, Insm1 suppresses *Hes1* expression by direct binding of a *cis*-regulatory sequence (Jia et al., 2015).

As described in the previous section, NEBs act as airway O_2_ sensors by expressing O_2_-sensing molecular complexes and by responding to hypoxia in adults (Youngson et al., 1993). Thus, environmental O_2_ could also be involved in the differentiation of PNECs during fetal lung development. Supporting this idea, *ex vivo* wild-type lung culture in hypoxic conditions can lead to decreased PNEC differentiation compared to normoxia, whereas the impact of hypoxia on differentiation is restricted to the early stages of lung development (McGovern et al., 2010). In addition, PNECs express prolyl hydroxylase domain enzymes (PHDs), which respond to hypoxia by catalyzing the hydroxylation of hypoxia-inducible factor 1 (HIF-1; Box 1), thus stabilizing it (Semenza, 2009). Importantly, loss of PHDs provokes a PNEC hyperplasia phenotype (Pan et al., 2012, 2016). The role of HIF-1 in PNEC differentiation could be another interesting topic for future studies.

These recent studies revealed the critical signaling circuits that regulate PNEC differentiation in development. Nonetheless, the initial inducer of *Ascl1* expression in the earliest progenitor cells remains unidentified. Further research is still required to provide a complete picture of the mechanisms of PNEC differentiation.

### Migration of PNECs in NEB development

Following solitary PNEC differentiation in the developing lung via Notch-mediated lateral inhibition, these further cluster into nodal and internodal NEBs (Avadhanam et al., 1997; Cutz et al., 1978; Hoyt et al., 1990; Noguchi et al., 2015) (Fig. 1). However, classical two-dimensional analysis – such as thin-tissue slice immunohistochemistry – does not reflect the complex 3D branching structure of the airway. 3D imaging of fetal lungs at single-cell resolution solved this problem, and revealed that nodal NEBs grow predominantly to larger sizes and in stereotypic locations along the airway (Noguchi et al., 2015). By contrast, internodal NEBs can be more often observed in the distal developing lung, and distal internodal NEBs are composed of fewer PNECs. Because nodal NEBs are the most represented type of PNEC cluster in the adult lung, a dynamic process such as cell migration or selective cell death has been speculated to be involved during NEB development.

**Fig. 3.**
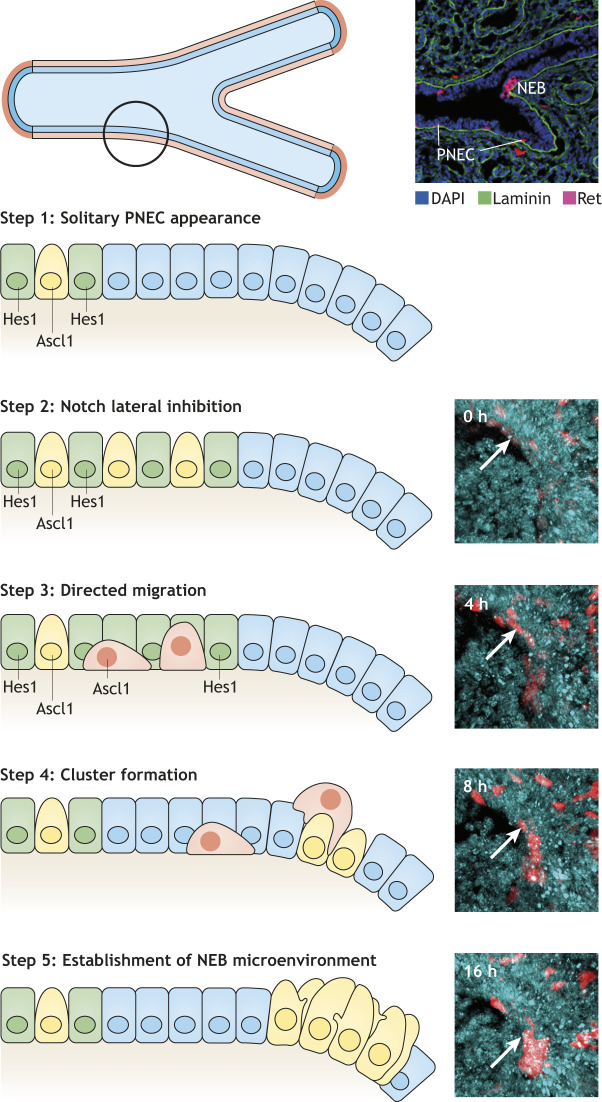
**NEB development.** Top left: branching airway. The circle indicates the area illustrated in the schematics below. Top right: developing airway of a fetal mouse at E14.5 [blue, 4′,6-diamidino-2-phenylindole (DAPI); green, laminin of basal membrane; magenta, Ret-expressing PNECs]. The development of an NEB is a stepwise process. Step 1: PNECs differentiate in the developing airway. Step 2: many PNECs appear while keeping distance between each other by Notch-mediated lateral inhibition (see Fig. 2). Step 3: PNECs detach from neighboring cells and migrate to the airway bifurcation site. Step 4: PNECs form clusters at the bifurcation site. Step 5: a primitive NEB microenvironment develops. Shown on the right are snapshots from time-lapse imaging of the developing murine airway (red, PNECs; blue, epithelial cells; arrows indicate migrating PNECs). These images are reproduced and modified from Noguchi et al. (2015) under the terms of the CC-BY 4.0 license.

Two groups independently performed live-cell imaging of developing PNECs and NEB formation (Kuo and Krasnow, 2015; Noguchi et al., 2015) to address these questions. Kuo and Krasnow (2015) performed embryonic lung slice culture to reveal that individual solitary PNECs migrate and aggregate at airway bifurcation sites (Fig. 3). PNECs exhibit migrating cell features, such as apical extensions, fibroblast-like morphology and thin cellular extensions converging toward the basal membrane during migration. Noguchi et al. (2015), using 3D time-lapse imaging of a whole embryonic lung lobe, reported directional migration of solitary PNECs toward the branching sites and subsequent formation of nodal NEBs. Some PNECs performed a periodical expansion-contraction movement of their protrusive structures during the migrating period. High-resolution 3D imaging further detected that the solitary PNECs remain attached to the basal membrane even when they extended their cytoplasmic processes, supporting the model that PNECs migrate on the basal membrane in a directional fashion, from distal areas to branching points. When solitary PNECs meet other PNECs, they dynamically change their behavior, including repeated transient contacts with other PNECs, resting, and extending away. Eventually, solitary PNECs enter into the packed PNEC cluster (Kuo and Krasnow, 2015).

How do PNECs acquire their migration capability when the progenitors are connected with neighboring epithelial cells? A recent report showed that Slit–Roundabout (ROBO) signaling (Box 1) is responsible for PNEC clustering by regulating migration activity. PNECs express the ROBO receptor, and some, but not all, express its ligand Slit1/2. ROBO and Slit mutant mice exhibit a reduced number of NEBs and an increase in solitary PNECs, indicating that the Slit–ROBO axis regulates PNEC clustering into NEBs. Additionally, the same report showed that Slit acts as an attraction signal for PNECs and that the Slit–ROBO axis regulates not only the migration but also the maintenance of homophilic PNEC adhesion (Branchfield et al., 2016).

PNEC migration reoccurs in the adult lung epithelium in case of severe injury. Ouadah et al. (2019) describe that adult lung PNECs migrate during the regeneration process of epithelial tissue in mice. Few solitary PNECs spread and locate tens to hundreds of micrometers away from NEBs during the first week after naphthalene-induced lung injury. Dispersed PNECs show cytoplasmic extensions, suggesting cells' detachment from the cluster and subsequent migration. These morphological alterations might reflect the reactivation of migratory molecular mechanisms during regeneration that further live-cell-imaging studies could confirm.

Several intriguing questions have been raised from the identification of this novel mode of epithelial directional migration. Whether Slit is the sole chemoattractant for directional migration or requires additional ones remains unclear; for instance, tissue structure might also be involved. Moreover, the mechanisms by which solitary PNECs recognize conspecific PNECs and change their behavior (e.g. pausing, direction changing) still need additional clarification. Further investigations will answer these questions and achieve a comprehensive understanding of PNEC migration.

## Are PNECs a stem cell niche or stem cell population?

### NEB as a stem cell niche

**Fig. 4.**
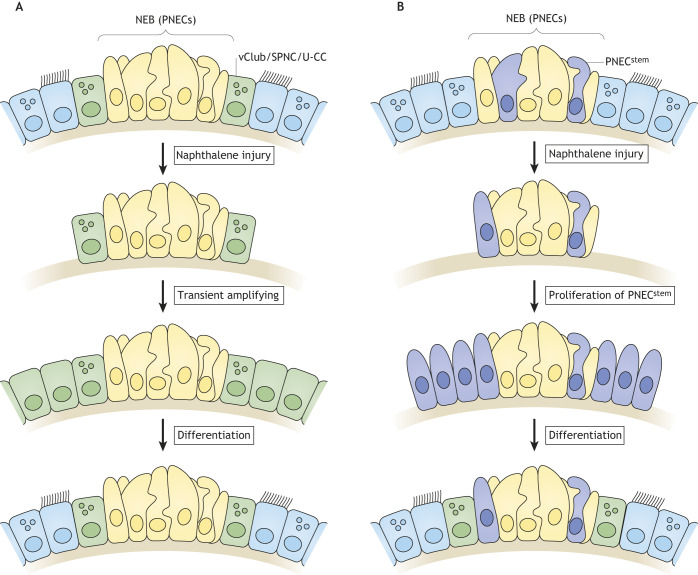
**The NEB microenvironment can foster two regeneration modes.** NEB-mediated epithelial regeneration in the naphthalene injury-repair model occurs in two modes: (A) the variant club cells (vClub)/SSEA-1^+^, peri-pNEB, N1ICD^+^, CC10^–^ cells (SPNC)/uroplakin-3a^+^ club cells (U-CC), which reside next to PNECs, act as transient-amplifying cells after injury; (B) the rare PNECs (PNEC^stem^), which reserve stem cell potential, contribute to tissue regeneration after injury.

The airway epithelium has regenerative capabilities upon acute damage by external pollutants and tobacco smoke, as well as upon viral infection such as influenza and severe acute respiratory syndrome coronavirus 2 (SARS-CoV-2). Once the airway tissue is injured, this stimulates the self-renewal of tissue stem cells for damage repair. The responsibility of adult tissue stem cells for tissue regeneration has motivated extensive investigation of adult airway epithelium stem cells in the past decades. This research advocated the NEB microenvironment as the potential stem cell source and niche. Classically, adult tissue stem cells are slow cycling, retaining the bromodeoxyuridine (BrdU) label for an extended period; hence, are named ‘label-retaining cells’ (LRCs). Airway epithelial LRCs were found at the cartilage–intercartilage junctions, where solitary steady-state PNECs frequently reside (Borthwick et al., 2001). The naphthalene-induced acute epithelial injury repair model revealed that epithelial recovery preferentially occurs around nodal NEBs (Stripp et al., 1995; Giangreco et al., 2009). In this model, both club cells (Box 1) and PNECs proliferate in NEBs during regeneration (Reynolds et al., 2000). This study discovered a peculiar subpopulation of club cells next to PNECs at the bronchioalveolar–duct junctions, termed variant club (vClub) cells, which act as transient-amplifying cells (Reynolds et al., 2000) (Fig. 4A). The majority of club cells catalyze the conversion of naphthalene into the highly toxic naphthalene 1R,2S-oxide by cytochrome P450-2F2, resulting in necrosis (Buckpitt et al., 1995; Mahvi et al., 1977). However, vClub cells are cytochrome P450-2F2 deficient and thus unable to metabolize naphthalene, which renders them resistant to naphthalene-induced injury and probably indicates a role in airway epithelium repair (Reynolds et al., 2000; Hong et al., 2001).

How do PNECs communicate to vClub cells within NEBs? The application of extracellular potassium rapidly mobilizes intracellular Ca^2+^ in PNECs, followed by a delayed increase of intracellular Ca^2+^ in a club-like cell population (De Proost et al., 2008). PNEC depolarization evokes Ca^2+^-mediated ATP release and activates these club-like cells via P2Y purinergic receptors, suggesting a functional coupling between PNECs and vClub cells (Schnorbusch et al., 2013). By contrast, a mild injury model induced with lipopolysaccharide (LPS; Box 1) selectively evokes oscillations in Ca^2+^ levels and proliferation of club-like cells, but with no PNEC activation (Verckist et al., 2018), suggesting that PNECs specifically respond to severe injury – such as that from naphthalene – and may supply niche factors for surrounding cells.

Notch and Dll ligands regulate the establishment and maintenance of the NEB microenvironment. In addition to vClub cells, a vClub-like population locates next to NEBs in the fetal lung. Our own research group and Guha et al. (Morimoto et al., 2012; Guha et al., 2012) showed that SSEA-1^+^, peri-NEB, Notch1 intracellular domain^+^ (N1ICD^+^), CC10^–^ (also known as SCGB1A1^–^) cells (SPNCs) and uroplakin-3a^+^ club cells (U-CCs) locate around NEBs during development, similar to vClub cells. The loss of Notch receptors reduces the number of SPNCs/U-CCs, suggesting that SPNCs/U-CCs require Notch signaling for their maintenance (Morimoto et al., 2012). Remarkably, knocking out the Dll ligand in PNECs expands Notch-activated SPNC/U-CC domains in association with NEB enlargement (Stupnikov et al., 2019). Conversely, selective ablation of SPNC cells does not affect the number of PNECs, suggesting that SPNC cells do not regulate PNECs through non-cell-autonomous suppression (Noguchi et al., 2015).

### PNECs as epithelial stem cells

For a long time, PNECs have been considered as an injury-resistant population capable of maintaining the NEB-associated vClub cells/SPNCs/U-CCs during development and regeneration, but not as stem cells themselves. Nonetheless, given their label retention, researchers have not yet excluded the stem cell potential of PNECs. A combination of PNEC-lineage tracing using *Cgrp^C^^reER^* mice and epithelial injury experiments revealed a certain degree of cellular plasticity during regeneration (Song et al., 2012). PNECs can differentiate into club and ciliated cells following airway epithelial injury. However, the authors did not exclude the possibility that the cellular plasticity observed might result from the activation of the CGRP promoter in regenerating club cells, as well as from the induction of CreER by residual tamoxifen due to insufficient washout (Hogan et al., 2014). This was solved by avoiding unintended labeling of regenerating Club cells. Naphthalene injury was performed 4 weeks after the last dose of tamoxifen, which clarified that non-PNECs do not contribute to the *Cgrp^C^^reER^* lineage. Furthermore, Notch – but not Hedgehog or Yap – signaling regulates the transdifferentiation of PNECs into club and ciliated cells during regeneration (Yao et al., 2018). In addition, Notch enhancement seems to induce the dedifferentiation and proliferation of PNECs after injury (Yao et al., 2018).

The heterogeneity of tissue stem cells is an important research topic, because a few – but not all – tissue stem cells can contribute to tissue homeostasis, including that of the lung (Zacharias et al., 2018). A recent report determined that rare PNECs (PNEC^stem^) – two to four cells per cluster of 20–30 cells – retain stemness and can contribute to tissue regeneration after injury. PNEC^stem^ undergo reprogramming, eventually converting into ‘transitional’ cells through Notch signaling (Fig. 4B). Transitional cells further reprogram to either club, ciliated, alveolar type 2 or stromal cells. Although Notch seems to be necessary and sufficient for PNEC deprogramming, reprogramming requires additional signals (Ouadah et al., 2019). For example, Yao et al. (2018) reported that epigenetic regulation by polycomb repressive complex 2 (PRC2; Box 1) deprograms PNECs under Notch signaling. Although PNECs act as stem cells during the regeneration process in the severe injury murine model, they contribute little to the epithelial regeneration in the usual environment. Genetic ablation of PNECs in mice seems not to affect homeostasis nor regeneration of the airway (Song et al., 2012), because alternative stem cell populations in the airway epithelium – such as club and basal cells – can take over cell replenishment.

Future work will further characterize the molecular basis for stemness maintenance and post-injury PNEC^stem^ activation. Furthermore, additional bona fide markers of PNEC^stem^ would help us to thoroughly understand the PNEC^stem^ and niche factors within NEBs. Because the hematopoietic stem cell system is regulated by direct innervation and neurotransmitter release from neurons (Agarwala and Tamplin, 2018), the role of neural regulation within NEB niches could be another fascinating topic of interest.

## SCLC derived from PNECs

PNECs are one of the potential origins of SCLC, sometimes called oat cell cancer. SCLC, an aggressive tumor with a poor prognosis, accounts for 10–15% of all lung cancers and represents the most common form of neuroendocrine lung cancer.

The similarity between SCLC and PNECs has been recognized because of their cellular morphology and neurosecretory-type granule content (Bensch et al., 1968). Several pioneering studies on transgenic mice demonstrated that PNECs might be the origin of SCLCs. Meuwissen et al. (2003) described the first SCLC model mouse, an adeno-Cre conditional double-knockout (DKO) of transformation-related protein 53 (Trp53) and retinoblastoma protein 1 (Rb1) within adult airway epithelial cells, which developed aggressive lung tumors, which appeared morphologically and immunophenotypically similar to SCLC. This model recapitulates the typical SCLC genotype, as comprehensive genomic analyses show bi-allelic losses of *TP53* and *RB1* in 100% and 93% of SCLC patients, respectively (George et al., 2015). Some PNEC hyperplasia was observed in the DKO model, fueling the hypothesis of SCLC development from PNECs (Meuwissen et al., 2003). A validation study using adeno-associated vectors to deplete Trp53 and Rb1 showed that CGRP-Cre, which targets PNECs, efficiently gave rise to SCLC, whereas vectors targeting type II alveolar cells (Box 1) had lesser efficiencies. These results identify PNECs as the predominant cell of origin of SCLC (Sutherland et al., 2011). Crossing *Cgrp^CreER^* PNEC lineage-tracing mice with the above-described DKO further confirmed that SCLC originates from differentiated PNECs. Moreover, loss of PTEN – a negative regulator of PI3K signaling and found mutated in 6% of SCLC patients – in *Trp53*- and *Rb1*-knockout mice sharply accelerated SCLC development (Song et al., 2012). These reports demonstrate that oncogenic mutations in *p53*, *Rb1* and *PTEN* in PNECs can trigger SCLC in mice and humans.

Do SCLCs emerge from a specific subpopulation of PNECs, given the heterogeneity of PNEC stem cell potential? PNEC^stem^ cells – a Notch-active subpopulation – proliferate significantly more than other PNECs following *Trp53* and *Rb1* deletion. In addition, *Trp53*/*Rb1*-deficient PNEC^stem^ cells fail to re-enter a quiescent state. The loss of *Trp53*/*Rb1* also enhances PNEC migration, potentially explaining the role of *Trp53*/*Rb1* in the pathognomonic features of SCLCs, such as their strong metastatic ability and aggressiveness (Ouadah et al., 2019).

Comprehensive genomic profiling of human SCLCs revealed several additional oncogenes promoting PNEC transformation into SCLC. Inactivating mutations in Notch family genes were detected in 25% of the cases. The majority of SCLC tumors had a low Notch pathway activity, including high expression of the Notch-inhibiting genes *ASCL1* and *DLK1* (George et al., 2015). Supporting this idea, conditional activation of the Notch pathway by N2ICD (the active form of Notch2) overexpression significantly suppressed the progression of tumors in *Trp53*, *Rb1* and *p130* (also known as *Nolc1*) triple-knockout mice, indicating a negative role of Notch signaling in SCLC development (George et al., 2015). Thus, Notch pathway-related factors are possible therapeutic targets for SCLC. Based on the accumulating evidence suggesting this, Augert et al. (2019) explored epigenetic modulation of Notch transcription. Lysine-specific histone demethylase 1 (LSD1; also known as KDM1A; Box 1) suppresses the expression of *NOTCH1*. The authors showed that an LSD1 blocker, ORY-1001, activates Notch signaling and inhibits tumor growth.

There are two opposite Notch-targeted therapeutic approaches for SCLC: Notch activation for suppressing tumor progression and Notch inhibition for reducing its heterogeneity. Lim et al. (2017) reported that a fraction of SCLCs express high levels of *Hes1*, indicating that Notch is activated in this SCLC subtype. This Notch-active population – called non-neuroendocrine (NE) SCLC – expresses lower levels of neuroendocrine markers. Endogenous Notch activation switches NE SCLC to the non-NE type through the expression of *Rest*, a transcription repressor; this means that levels of Notch activation may reflect the heterogeneity of SCLC cells. Non-NE SCLC cells are less proliferative and more chemoresistant than NE SCLC cells. This subpopulation further secretes the growth factor midkine to promote NE SCLC cells' growth. Thus, Notch-mediated diversification within SCLC cells is one of the key events for the malignant transformation of this cancer.

Inhibiting Notch avoids the occurrence of SCLC heterogeneity. The combination of tarextumab – which inhibits Notch2/3 – and carboplatin/irinotecan suppresses SCLC tumor growth and delays relapse in SCLC allograft models, as well as in patient-derived xenografts in mice. A phase 1b clinical trial (NCT01859741) indicated that tarextumab treatment improved overall and progression-free survival in patients with advanced SCLC whose tumors expressed elevated levels of Notch genes; however, the above-mentioned combination therapy was not effective in a phase 2 follow-up with a larger cohort (Lim et al., 2017). Therefore, the Notch inhibition strategy might be beneficial in the early stages of SCLCs, when heterogeneity first arises.

## PNECs in lung pathogenesis

**Fig. 5.**
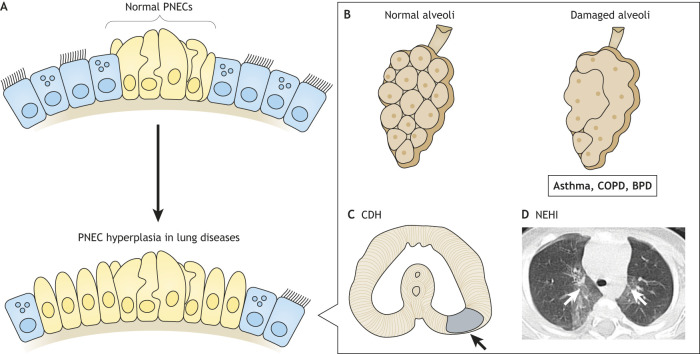
**PNEC hyperplasia in lung diseases.** (A) PNEC hyperplasia is an abnormal expansion of PNECs and is associated with several lung diseases. (B) Pulmonary emphysema occurs upon alveolar septal destruction in asthma, chronic obstructive pulmonary disease (COPD) and bronchopulmonary dysplasia (BPD). (C) Schematic representation of inadequate closure of the pleuroperitoneal membrane in congenital diaphragmatic hernia (CDH) patients (arrow). (D) Chest high-resolution computed tomography in neuroendocrine hyperplasia of infancy (NEHI); characteristic ground-glass opacities can be observed (arrows). This panel was reproduced and modified from Popler et al. (2010). This image is not published under the terms of the CC-BY license of this article. For permission to reuse, please see Popler et al. (2010).

NEB morphology and functions relate to several intractable human pulmonary diseases, including common chronic lung diseases such as asthma and COPD. Furthermore, excessive PNECs are found in congenital diseases such as bronchopulmonary dysplasia (BPD), congenital diaphragmatic hernias (CDH) and neuroendocrine hyperplasia of infancy (NEHI) (Fig. 5). This PNEC hyperplasia could be linked to the sensory and regenerative functions of this cell population (see ‘PNECs as sensory transducers’ and ‘Are PNECs a stem cell niche or stem cell population?’ sections). In this section, we overview the current evidence of the association between PNECs and human diseases.

### BPD

BPD is a chronic lung disease in premature infants who were treated with artificial mechanical ventilation at high O_2_ concentrations. BPD patients suffer from long-term lung dysfunction, representing a heavy burden on medical care services (El Mazloum et al., 2014; Northway et al., 1967). BPD patients generally show intense airway inflammation, lung fibrosis, disrupted alveolarization and thickened interalveolar septa with reduced gas exchange efficiency (Husain et al., 1998). Bombesin-immunoreactive PNECs appear more frequently in BPD patients than in control infants (Johnson et al., 1982). Furthermore, post-surfactant BPD is associated with PNEC hyperplasia and increased proliferation of surrounding cells, indicating an altered NEB microenvironment in BPD (Cutz et al., 2007).

Does PNEC hyperplasia lead to BPD phenotypes? Elevated bombesin-like peptide (BLP)/gastrin-releasing peptide (GRP) (Box 1) levels in PNECs have been proposed as an etiological agent of and a potential therapeutic target for BPD. Cumulative observations of infants revealed that urinary BLP elevation at 1–4 days after birth associates with a tenfold increase in BPD risk (Cullen et al., 2002). Intratracheal administration of BLPs into mice induced alveolar myofibroblast proliferation and increased alveolar wall thickness, typical symptoms of human BPD. These BPD-like phenotypes can be relieved by the deletion of the murine GRP receptor (*Grpr*) gene, suggesting that BLP overproduction in PNECs could be one of the causes of BPD through the activation of GRPR signaling (Ashour et al., 2006).

Why do PNECs in BPD patients produce excessive amounts of BLPs? Because intermittent hypoxic episodes associate with BPD (Martin et al., 2015), PNEC hyperplasia is most likely induced by hypoxia. Moreover, the majority of PNECs in post-surfactant BPD express *HIF1A*, which is suppressed in the normal lung (Cutz, 2015), and loss of *PHD* induces PNEC hyperplasia (see ‘Development of PNECs and NEBs’ section). Further clarification of the mechanisms behind hypoxia-induced PNEC hyperplasia will be necessary to find a therapeutic target for BPD.

### COPD

COPD is a common respiratory disease – the third leading cause of death globally – characterized by chronic airflow limitations and persistent respiratory symptoms, such as dyspnea and cough. Airflow is limited by a multitude of small-airway disruptive phenomena along with emphysema (Box 1), which is mainly triggered by cigarette smoking (https://goldcopd.org/; Hogg and Timens, 2009). COPD patients have more PNECs in their airway compared to healthy subjects (Gu et al., 2014). Reflecting this observation, higher BLP levels have been detected in the bronchoalveolar lavage fluid of cigarette smokers (Aguayo et al., 1989). Even healthy smokers show BLP elevation, suggesting that PNEC hyperplasia, which is often associated with increased sensitivity to chemical stimuli (Tashkin et al., 1996), could occur before smokers develop actual COPD. PNEC hyperplasia could cause this chemical hyperresponsiveness and exaggerated reaction by peptide secretion, which may lead to COPD. Human PNECs express OR2W1, a member of the olfactory receptor family, which responds to inhaled volatile chemicals, eventually releasing 5-HT to induce airway smooth muscle contraction (Gu et al., 2014), and may therefore be the key receptor for chemical stimuli responses in COPD. Thus, chemoreceptors on PNECs and their downstream signaling could become future therapeutic targets for managing diseases associated with airway hypersensitivity in COPD.

### CDH

CDH is a congenital disease characterized by the loss of diaphragm integrity – most often a discontinuity – causing displacement of the abdominal content into the thoracic cavity (Kardon et al., 2017). A meta-analysis of CDH cases showed a prevalence of 1/4000 births. CDH is often accompanied by pulmonary hypoplasia and pulmonary hypertension (Pober, 2007). Clinical case studies show an increase in PNECs/NEBs in the lung of CDH infants compared to newborns with lung hypoplasia due to other causes (Ijsselstijn et al., 1997). A recent exome-sequencing study of CDH patients found that point mutations in SLIT and ROBO genes associate with CDH (Longoni et al., 2014). Interestingly, genetic ablation of *Robo1* and *Robo2* in mice leads to organ misplacement and diaphragm malformation, recapitulating CDH (Domyan et al., 2013). *Robo1*- and *Robo2*-deficient mice have few NEB clusters, but many solitary PNECs distributed in the airway epithelium. This disruption of PNEC clustering increases the number of immune cells in the airway lumen while also simplifying alveoli. Hence, clustered PNECs could play a role in decreasing the number of immune cells in the naïve neonatal lung (Branchfield et al., 2016). *Robo1*- and *Robo2*-deficient mice closely resemble human CDH phenotypes, except for the increase in PNECs. Integrating work in this mouse model with further analyses using induced PNECs from CDH patient-derived induced pluripotent stem cells (iPSCs) would clarify whether PNEC hyperplasia could be linked to genetic defects in CDH patients.

### NEHI

NEHI is a rare pediatric lung disease characterized by tachypnea, retractions, crackles and hypoxemia (Deterding et al., 2001). Intense hyperplasia of bombesin^+^ PNECs has been consistently observed in bioptic specimens of NEHI patients' lungs (Deterding et al., 2005). Although the cause of NEHI remains poorly understood, this disease shows an apparent autosomal-dominant inheritance pattern. A genetic study in a familial cohort identified a G-to-T transversion in codon 191 of *NKX2.1* (Box 1), resulting in the substitution of leucine for arginine (Young et al., 2013). Before this study, NEHI was considered a childhood-specific disorder, gradually improving over time; however, this family cohort unveiled lifelong pulmonary abnormalities (Nevel et al., 2016). Murine genetic models might experimentally reproduce NEHI. Genetic ablation of *NKX2.1* leads to PNEC loss, suggesting that *NKX2.1* plays a crucial role in PNEC physiology (Li et al., 2013). A more accurate NEHI murine model, such as an *Nkx2-1* L19G mutant, would help shed light on the etiology of NEHI and clarify whether PNEC hyperplasia is the primary causative mechanism or a secondary consequence.

In summary, PNEC hyperplasia often occurs in pulmonary diseases, probably contributing to their pathogenesis. Controlling the numerical plasticity of PNECs through pharmacological agents could be beneficial for long-term clinical outcomes of chronic pulmonary diseases.

## New experimental tools

Owing to the rarity of PNECs in the lung, PNEC research has been difficult. Recent improvements enable the visualization of this rare cell population through 3D studies of the branching airway at single-cell resolution, the determination of rare subpopulations within PNECs via single-cell transcriptomics, the estimation of clonal cell expansion using *in vivo* lineage tracing, and the tracking of the biology-to-pathology progression through a protocol to generate human PNECs from human pluripotent stem cells (hPSCs). Here, we describe the advanced technologies for understanding the biological features of PNECs (Table 1).

**Table 1.**
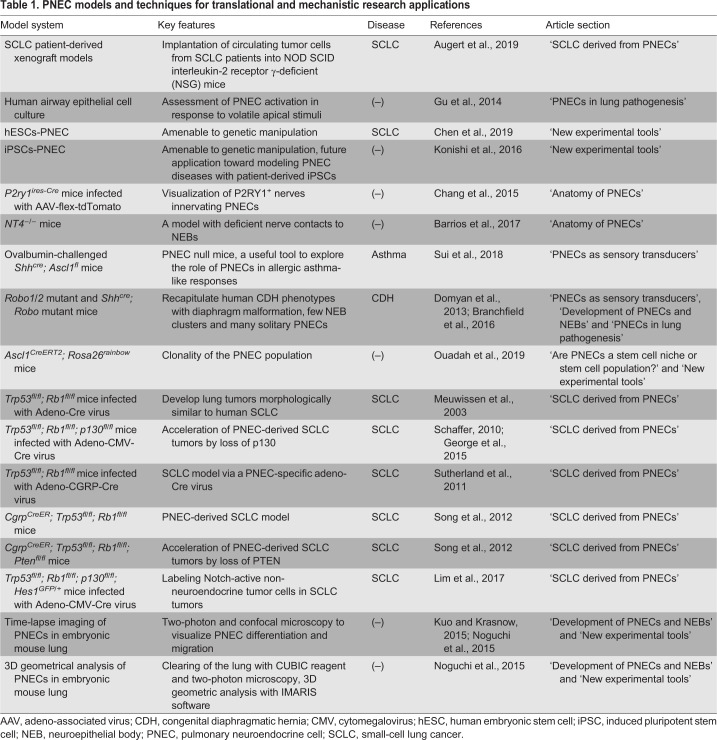
**PNEC models and techniques for translational and mechanistic research applications**

### Induced human PNECs from pluripotent stem cells

Several recently developed protocols can generate various human lung cell types from hPSCs. Chen et al. (2019) induced PNECs from human embryonic stem cells (hESCs) by recapitulating the developmental environment step-by-step *in vitro* using the protocol described by Huang et al. (2015), sequentially inducing definitive endoderm, anterior foregut endoderm and then lung progenitor cells by exposing cells to growth factor cocktails. Notch signaling inhibition drives the differentiation of progenitor cells into PNECs (see ‘Development of PNECs and NEBs’ section). Consistently, Notch inhibitors promote the differentiation of hESC-derived lung progenitors into PNECs (8.9±1.9% of the total cells) (Chen et al., 2019). hPSC-derived PNECs would be useful to reveal the underlying mechanisms of PNEC development in humans, possibly leading to an improved understanding of the causal link between congenital pulmonary diseases and genetic alterations, e.g. NEHI with a point mutation in *NKX2.1*.

Intriguingly, *RB1* knockdown in hPSC-derived PNECs produces more CGRP-expressing cells with a similar transcriptomic profile to that of SCLC. Furthermore, *TRP53/RB1* DKO allows xenografted cells to form early-stage tumors resembling SCLCs (Chen et al., 2019). Interestingly, 3D spheroids of iPSC-derived proximal airway epithelial progenitors can also differentiate into PNECs upon Notch inhibition (Konishi et al., 2016). Generating SCLCs from patient-derived iPSCs could elucidate the association between patient-specific genomic backgrounds and tumor initiation.

### Deep-tissue 3D and 4D imaging

Conventional histological image analysis is broadly used to collect primary positional information on PNECs and to enable histopathological diagnosis. However, the information is limited in the *x*–*y* plane due to its narrow observation range in the *z*-axis. Alternative methods for deep-tissue imaging should be developed to capture the bona fide distribution of PNECs in the 3D branching structure of the airway. Two-photon microscopy overcomes the limitation of confocal microscopy by reducing the sensitivity to light scattering and realizes the vast imaging depths in intact and fixed tissues (Helmchen and Denk, 2005).

**Fig. 6.**
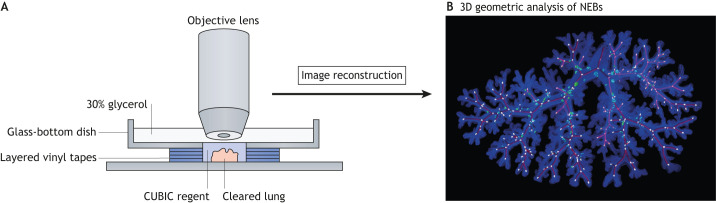
**Generation of a high-resolution 3D image of NEBs by two-photon microscopy.** (A) The isolated fetal mouse cranial lung lobe is cleared with CUBIC, a hydrophilic tissue-clearing reagent. The cleared specimen is placed in a custom chamber and the whole lobe is imaged with a two-photon microscope using a ×25 objective lens. (B) Geometric computational analysis of the high-resolution 3D image reveals the stereotypic distribution of nodal NEBs (green/cyan). The 3D structure of the entire airway epithelium is visualized (blue). The central lines of the bronchial lumen structure (purple) are drawn using IMARIS filament tracing. The panel is reproduced and modified from Noguchi et al. (2015) under the terms of the CC-BY 4.0 license.

Our group previously described a methodology for four-dimensional (4D; 3D plus time) imaging, which enables us to track PNEC migration with live imaging (Noguchi et al., 2015) (Fig. 6). We combined air-liquid culture of murine embryonic pulmonary lobe with inverted two-photon microscopy, using highly sensitive detectors to reduce phototoxicity by minimizing the excitation and damage to the cultured lobes. We also took advantage of computational drift correction to compensate for any movement of the growing lobes, which is necessary for tracking the same PNEC at different time points. However, the current method is not sufficient to examine the subcellular structure of migrating PNECs, such as cytoskeleton dynamics. Further methodological improvements could visualize it in 4D to uncover the detailed mechanisms of their unique migration modes.

Lung tissue is complex enough to interfere with emission/excitation lights because of different refractive indices (RIs). Recently developed clearing reagents intend to homogenize the differential RIs between specimens and imaging media (Ueda et al., 2020). Such clearing reagents improve visibility in the deep area of the embryonic airway epithelium while permitting single-cell imaging of PNECs in the intact lobe. Moreover, the resulting high-resolution 3D image enables geometric computational analysis of PNEC distribution (Noguchi et al., 2015) (Fig. 6). Optimization of the clearing reagent CUBIC also achieved a 3D pathological profiling of cancer lung metastases at single-cell resolution (Kubota et al., 2017). Thus, improvement in deep-tissue 3D imaging would help to accurately map the spatial alterations in PNECs occurring during lung disease.

### Rainbow mouse and scRNA-seq

The multicolor chimeric ‘rainbow’ mouse is a useful tool to analyze the clonality, lineage and heterogeneity of proliferating cells. Cells in the rainbow mouse carry four different fluorescent protein complementary DNAs in three sets of *loxp*-flanked sites at the *Rosa26* genomic locus (Rinkevich et al., 2011). Cre recombinase randomly removes three *loxp* sites, inducing the expression of one out of every four colors. This multicolor lineage tracing increases the accuracy of statistical and clonal analyses compared to conventional lineage-tracing methods (Rinkevich et al., 2011). Multicolored PNECs are detected in a single NEB of *Shh^CreERT2^;* *Rosa26^rainbow^* mice, indicating that PNECs have different origins and do not clonally proliferate during development (Kuo and Krasnow, 2015). Unlike in development, sizable clonal PNEC patches populate the regenerating epithelium of adult *Ascl1^CreERT2^;* *Rosa26^rainbow^* mice. Namely, a single PNEC^stem^ within an NEB has clonal outgrowth potential and restores the damaged epithelium after injury. Computational simulations based on empirical fitting revealed that 17% of parental PNECs are PNEC^stem^ and generate 1–3 daughter cells each (Ouadah et al., 2019).

Aside from multicolor lineage tracing, scRNA-seq is also a relevant rising technology to investigate cellular heterogeneity within a tissue, providing genome-wide expression profiles of individual cells (Treutlein et al., 2014). Noteworthily, scRNA-seq of PNEC lineages and subsequent unbiased computational clustering identified previously unknown subpopulations, including reprogrammed PNECs in a transitional state (Ouadah et al., 2019). Single-cell trajectory analyses of PNECs at different time points during regeneration could further elucidate the detailed mechanisms and essential factors during the deprogramming and reprogramming processes. scRNA-seq is also applicable for the assessment of SCLC heterogeneity. In the hESC-derived SCLC *in vitro* model, scRNA-seq revealed that *RB1* knockdown generates three subpopulations with different transcriptional profiles (Chen et al., 2019). A combination of hPSC technology and scRNA-seq could find further heterogeneity generators and identify a therapeutic approach to reduce the heterogeneity of SCLCs to improve patient outcomes.

## Conclusions

We now stand at the dawn of a ‘PNEC renaissance’. The recent research milestones are prompting a renewed acknowledgment of the physiological importance and unique characteristics of PNECs. During the past few years, technological advances helped to develop comprehensive maps of each PNEC. These cutting-edge technologies enable a better understanding of PNECs as heterogenic cell populations and facilitate further functional explorations. These comprehensive data will accurately delineate the molecular basis of PNEC active directional migration during development and regulation of stem cell functions during the regeneration process. Further basic research on PNEC biology could elucidate the mechanisms behind pathological disorders of PNECs in several respiratory diseases, as well as their numerical dysregulation as in SCLCs. In addition, these studies could define PNEC plasticity and its potential as a therapeutic target. Therefore, murine models of PNEC diseases and PNECs differentiated from patient-derived iPSCs could become more relevant as research platforms in the future.

Several intriguing questions in PNEC research remain open. Notably, the physiological significance of their unique localization patterns, innervation and mechanosensing is still enigmatic. Future investigation aiming to address these questions could lead to a better understanding of the biological behaviors of PNECs along with their importance in physiology. The combination of basic studies on PNECs, together with the investigation of human etiology, will yield valuable clues for the identification of clinical targets in various lung disorders.
